# Supplementary material for: Enhancing cause of death prediction: development and validation of machine learning models using multimodal data across multiple health-care sites
Source: JAMIA Open. 2026 Jan 8;9(1):ooaf175. doi: 10.1093/jamiaopen/ooaf175 (PMC12924636; doi:10.1093/jamiaopen/ooaf175)
Supplement: ooaf175_Supplementary_Data [file ooaf175_supplementary_data.docx]

# **Supplementary Materials**

**Table S1 provides a summary of the data layers and features integrated into the model.**

| Data Layer | Features | Source | Feature Description |
| --- | --- | --- | --- |
| Structured Data | 2048 | EHR | - ICD-10-CM diagnosis and ICD-10-PCS mapped to Clinical Classification Software (CCS) - Laboratory tests mapped to LOINC groupers. - Outpatient and inpatient medications were aggregated to the Anatomical Therapeutic Chemical (ATC) 4 Classification level using RxNorm. - Vital signs and demographics were also included. |
| Unstructured Notes | 768 | EHR | - Language model Embeddings |
| Public data | 16 | Public data | - Separate variables for each of the Top 15 CoD & Other using NCHS 52 Ranked CoD groupers^25^ |

**Table S2.** Distribution of Top 15 CoD* at VUMC and MGB

| Top 15 CoD | VUMC | | MGB | |
| --- | --- | --- | --- | --- |
| Disease | Count | % | Count | % |
| Malignant Neoplasm | 4155 | 30.3% | 10961 | 31.5% |
| Diseases Of Heart | 2192 | 16.0% | 6445 | 18.5% |
| Covid19 | 1044 | 7.6% | 1135 | 3.3% |
| Unintentional Injuries | 1042 | 7.6% | 1270 | 3.6% |
| Cerebrovascular Disease | 612 | 4.5% | 1329 | 3.8% |
| Chronic Liver Disease and Cirrhosis | 364 | 2.7% | 324 | 0.9% |
| Chronic Lower Respiratory Disease | 353 | 2.6% | 1296 | 3.7% |
| Diabetes Mellitus | 306 | 2.2% | 769 | 2.2% |
| Nephritis, Nephrotic Syndrome, And Nephrosis | 194 | 1.4% | 852 | 2.4% |
| Influenza And Pneumonia | 188 | 1.4% | 693 | 2.0% |
| Septicemia | 157 | 1.1% | 525 | 1.5% |
| Intentional Self Harm | 153 | 1.1% | 118 | 0.3% |
| Parkinson Disease | 131 | 1.0% | 446 | 1.3% |
| Essential Hypertension and Hypertensive Renal Disease | 129 | 0.9% | 331 | 0.9% |
| Alzheimer’s Disease | 115 | 0.8% | 703 | 2.0% |
| All Other NCHS CoD | 2573 | 18.8% | 7642 | 21.9% |
| Total | **13708** | **100%** | **34839** | **100%** |

****CoD was defined using official death records from the NDI for VUMC and from state health departments (MA, CT, VT) for MGB.***

**Table S3.** Cross-Institutional Model Performance **^§^**

|  | **VUMC Trained Model** | | | | **MGB Trained Model** | |
| --- | --- | --- | --- | --- | --- | --- |
|  | **Full Model Tested VUMC (*S)** | **Full Model Tested MGB (*S)** | **Tested VUMC (**S+U)** | **Tested MGB (**S+U)** | **Full Model Tested MGB (*S)** | **Full Model Tested VUMC (*S)** |
| **Weighted AUC** | **0.86** | **0.49** | **0.9** | **0.51** | **0.8** | **0.55** |
| Malignant Neoplasm | 0.94 | 0.48 | 0.95 | 0.57 | 0.83 | 0.68 |
| Disease of heart | 0.99 | 0.47 | 0.86 | 0.48 | 0.91 | 0.51 |
| COVID19 | 0.81 | 0.55 | 0.86 | 0.47 | 0.74 | 0.41 |
| Unintentional injuries | 0.82 | 0.49 | 0.75 | 0.5 | 0.72 | 0.51 |
| Cerebrovascular disease | 0.67 | 0.35 | 0.89 | 0.4 | 0.89 | 0.53 |
| Chronic liver disease and cirrhosis | 0.88 | 0.53 | 0.78 | 0.51 | 0.78 | 0.44 |
| Chronic lower respiratory disease | 0.69 | 0.46 | 0.8 | 0.52 | 0.75 | 0.46 |
| Diabetes mellitus | 0.82 | 0.49 | 0.97 | 0.48 | 0.75 | 0.47 |
| Nephritis, nephrotic syndrome, and nephrosis | 0.96 | 0.54 | 0.86 | 0.5 | 0.68 | 0.55 |
| Influenza and pneumonia | 0.82 | 0.51 | 0.91 | 0.53 | 0.63 | 0.57 |
| Septicemia | 0.92 | 0.67 | 0.82 | 0.63 | 0.68 | 0.59 |
| Intentional self-harm | 0.73 | 0.43 | 0.89 | 0.48 | 0.88 | 0.41 |
| Parkinson disease | 0.89 | 0.5 | 0.76 | 0.53 | 0.63 | 0.63 |
| Essential hypertension and hypertensive renal disease | 0.67 | 0.6 | 0.79 | 0.44 | 0.81 | 0.51 |
| Alzheimer | 0.75 | 0.51 | 0.85 | 0.48 | 0.65 | 0.46 |
| All Other NCHS CoD | 0.77 | 0.51 | 0.98 | 0.47 | 0.73 | 0.52 |

**^§^**Confidence intervals are excluded as model degradation renders these calculations unwarranted. ****S****=Structured data* *****S+U****= Structured +Unstructured*

Table S 4. Overall weighted AUC AND AUC for Individual CoD at VUMC (S=Structured data S+U+PD= Structured +Unstructured +Public data)

|  | VUMC Trained and Tested | | | | MGB Trained and Tested | | | |
| --- | --- | --- | --- | --- | --- | --- | --- | --- |
| Disease | **Counts** | **S** | **S+U** | **S+U+PD** | **Counts** | **S** | **S+U** | **S+U+PD** |
| Overall Weighted AUC |  | 0.86 | 0.90 | 0.90 |  | 0.80 | 0.92 | 0.72 |
| Malignant Neoplasm | 4155 | 0.94 | 0.95 | 0.95 | 10961 | 0.83 | 0.90 | 0.81 |
| Diseases of heart | 2192 | 0.99 | 0.98 | 0.98 | 6445 | 0.91 | 0.98 | 0.71 |
| COVID19 | 1044 | 0.81 | 0.86 | 0.86 | 1135 | 0.74 | 0.89 | 0.87 |
| Unintentional injuries | 1042 | 0.82 | 0.86 | 0.89 | 1270 | 0.72 | 0.93 | 0.68 |
| Cerebrovascular disease | 612 | 0.67 | 0.75 | 0.76 | 1329 | 0.89 | 0.97 | 0.70 |
| Chronic liver disease and cirrhosis | 364 | 0.88 | 0.89 | 0.89 | 324 | 0.78 | 0.91 | 0.83 |
| Chronic lower respiratory disease | 353 | 0.69 | 0.78 | 0.78 | 1296 | 0.75 | 0.91 | 0.73 |
| Diabetes Mellitus | 306 | 0.82 | 0.80 | 0.84 | 769 | 0.75 | 0.93 | 0.72 |
| Nephritis, nephrotic syndrome, and nephrosis | 194 | 0.96 | 0.97 | 0.92 | 852 | 0.68 | 0.95 | 0.74 |
| Influenza and pneumonia | 188 | 0.82 | 0.86 | 0.84 | 693 | 0.63 | 0.93 | 0.65 |
| Septicemia | 157 | 0.92 | 0.91 | 0.90 | 525 | 0.68 | 0.94 | 0.62 |
| Intentional Self Harm | 153 | 0.73 | 0.82 | 0.82 | 118 | 0.88 | 0.97 | 0.47 |
| Parkinson disease | 131 | 0.89 | 0.89 | 0.89 | 446 | 0.63 | 0.94 | 0.82 |
| Essential hypertension and hypertensive renal disease | 129 | 0.67 | 0.76 | 0.76 | 331 | 0.81 | 0.96 | 0.62 |
| Alzheimer's Disease | 115 | 0.75 | 0.79 | 0.79 | 703 | 0.65 | 0.85 | 0.77 |
| All Other NCHS CoD | 2573 | 0.77 | 0.85 | 0.86 | 7642 | 0.73 | 0.88 | 0.61 |

**Comparative Analysis of Public Data Versus NDI/State Data on Causes of Death**


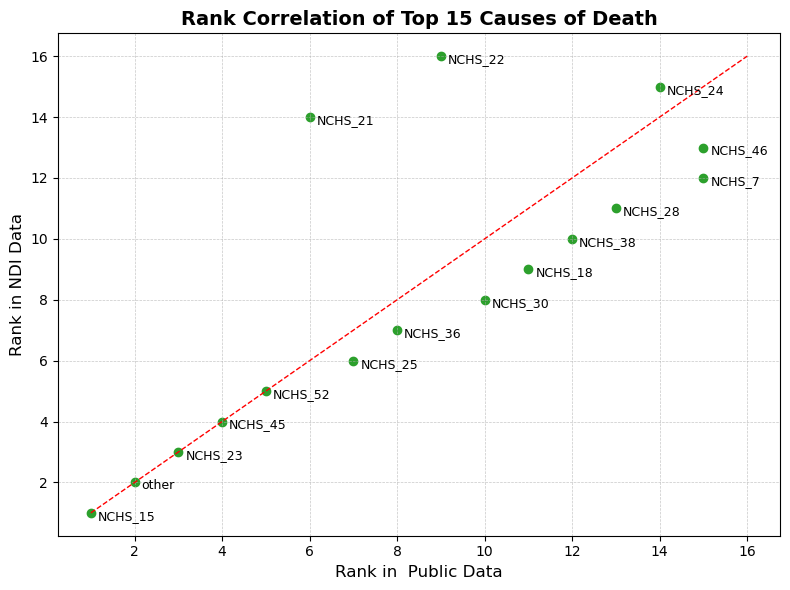


**Figure s1-A (VUMC)**


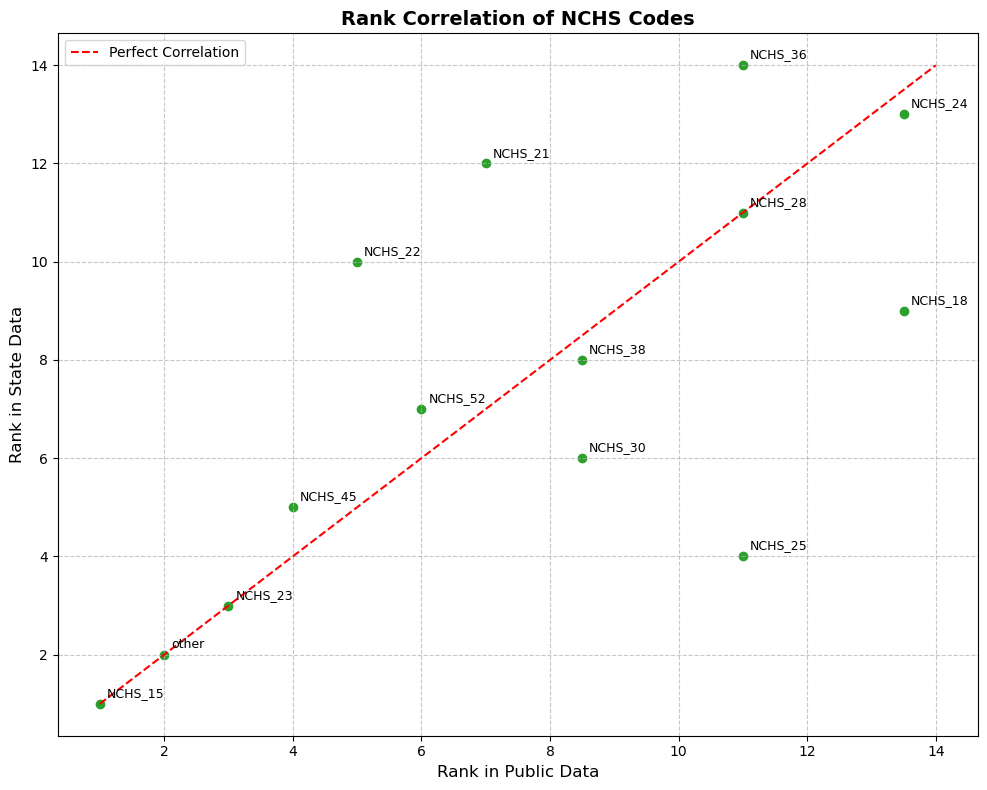


**Figure s1 B (MGB)**

Figure s1: Rank Correlation of Top 15 Causes of Death - Public Data vs NDI Data. (NCHS_15 (Malignant Neoplasm), NCHS_23 (Diseases of Heart), NCHS_52 (COVID-19), NCHS_45 (Unintentional Injuries), NCHS_25 (Cerebrovascular Disease), NCHS_36 (Chronic Liver Disease and Cirrhosis), NCHS_30 (Chronic Lower Respiratory Disease), NCHS_18 (Diabetes Mellitus), NCHS_38 (Nephritis, Nephrotic Syndrome, and Nephrosis), NCHS_28 (Influenza and Pneumonia), NCHS_21 (Parkinson’s Disease), NCHS_24 (Essential Hypertension and Hypertensive Renal Disease), NCHS_22 (Alzheimer’s Disease), and Other (Other Causes)).

**Graph Overview:**

**Figure A (VUMC):** The scatter plot illustrates a strong positive correlation between the ranking of causes of death in public and healthcare data, as shown by points closely following a diagonal trend line. This alignment indicates that more frequently mentioned causes of death in public data generally correspond to higher rankings in NDI data. However, a few deviations exist—such as Alzheimer’s Disease (NCHS_22) and essential hypertension (NCHS_24)—which rank higher in public data compared to healthcare records.

**Figure B (MGB):** Similar to VUMC, the scatter plot for MGB shows a positive correlation between the ranks in public and healthcare data, suggesting alignment in cause of death distributions across data sources. However, there are minor deviations from the trend line, such as certain conditions ranking higher in state data than in online mentions.

Together, Figures A and B suggest a strong rank correlation between public and institutional datasets across both VUMC and MGB, highlighting the consistency of cause of death distributions between public and healthcare records, with slight variations for specific conditions in each system.

**Spearman and Kendall Tau Correlations**.

**VUMC:** The correlation coefficients between public and healthcare data are high, with Spearman's ρ = 0.7785 (p = 0.0004) and Kendall's τ = 0.7113 (p = 0.0001), indicating statistically significant correlations. These values suggest a strong, consistent relationship between public reporting and healthcare data for CoD, implying alignment in cause-specific prevalence across sources.

**MGB:** Similarly, MGB demonstrates strong correlations, though slightly lower than VUMC, with Spearman's ρ = 0.6969 (p = 0.0056) and Kendall's τ = 0.5652 (p = 0.0058). These significant correlations reflect alignment between public perception and healthcare data at MGB, albeit with some variations in specific CoD rankings.

**S2: Supplementary: Feature selection results**

Feature selection methods—including Stability Selection, Random Forest Selection, and SelectFromModel—were applied to enhance model interpretability and potentially improve cross-site performance. Despite reducing dimensionality and enhancing interpretability, these methods did not substantially mitigate the performance degradation observed during cross-site evaluations.

Table S5 Feature Selection Methods and Performance

|  | **VUMC Trained and Tested** | | | | **VUMC Trained and MGB Tested** | | | |
| --- | --- | --- | --- | --- | --- | --- | --- | --- |
|  | **Original Model​** | **SFM** | **SS** | **RFS** | **Original** | **SFM** | **SS** | **RFS** |
| **Weighted AUC** | **0.86** | **0.87​** | **0.80​** | **0.87** | **0.49** | **0.49** | **0.51** | **0.62** |
| Malignant Neoplasm​ | 0.94​ | 0.95​ | 0.92​ | 0.94 | 0.48 | 0.53 | 0.55 | 0.65 |
| Disease of heart​ | 0.99​ | 0.98​ | 0.77​ | 0.98 | 0.47 | 0.34 | 0.54 | 0.82 |
| COVID19​ | 0.81​ | 0.80​ | 0.77​ | 0.82 | 0.55 | 0.51 | 0.41 | 0.53 |
| Unintentional injuries​ | 0.82​ | 0.81​ | 0.77​ | 0.82 | 0.49 | 0.55 | 0.47 | 0.48 |
| Cerebrovascular disease​ | 0.67​ | 0.63​ | 0.69​ | 0.7 | 0.35 | 0.65 | 0.63 | 0.4 |
| Chronic liver diseae and cirrhosis​ | 0.88​ | 0.89​ | 0.81​ | 0.89 | 0.53 | 0.42 | 0.44 | 0.52 |
| Chronic lower respiratory disease​ | 0.69​ | 0.68​ | 0.65​ | 0.66 | 0.46 | 0.52 | 0.54 | 0.43 |
| Diabetes mellitus​ | 0.82​ | 0.82​ | 0.75​ | 0.79 | 0.49 | 0.53 | 0.46 | 0.51 |
| Nephritis, nephrotic syndrome, and nephrosis​ | 0.96​ | 0.97​ | 0.92​ | 0.96 | 0.54 | 0.52 | 0.36 | 0.54 |
| Influenza and pneumonia​ | 0.82​ | 0.78​ | 0.75​ | 0.76 | 0.51 | 0.55 | 0.38 | 0.58 |
| Septicemia​ | 0.92​ | 0.91​ | 0.84​ | 0.92 | 0.67 | 0.55 | 0.49 | 0.66 |
| Intentional self-harm​ | 0.73​ | 0.75​ | 0.72​ | 0.73 | 0.43 | 0.54 | 0.49 | 0.51 |
| Parkinson disease​ | 0.89​ | 0.88​ | 0.87​ | 0.89 | 0.5 | 0.48 | 0.47 | 0.54 |
| Essential hypertension and hypertensive renal disease​ | 0.67​ | 0.65​ | 0.67​ | 0.7 | 0.6 | 0.34 | 0.47 | 0.45 |
| Alzheimer​ | 0.75​ | 0.74​ | 0.70​ | 0.75 | 0.51 | 0.52 | 0.46 | 0.51 |
| All Other NCHS CoD ​ | 0.77​ | 0.80​ | 0.73​ | 0.79 | 0.51 | 0.52 | 0.46 | 0.55 |
